# Coughing Can Be Modulated by the Hydration Status in Adolescents with Asthma

**DOI:** 10.3390/children9040577

**Published:** 2022-04-18

**Authors:** Alessandro Zanasi, Roberto Walter Dal Negro

**Affiliations:** 1 Lung Unit of the Italian Association for Studying Cough, AIST, 40100 Bologna, Italy; zanasi.tosse@gmail.com; 2 Research & Clinical Governance, 37124 Verona, Italy

**Keywords:** cough, asthma, adolescents, urine osmolality, dehydration, water

## Abstract

A lower thirst sensitivity frequently characterizes children and adolescents. The daily water intake can be frequently insufficient for the homeostasis and the integrity of their airway epithelium. Little is known about the real-life relationship between dehydration and coughing in young students with asthma. The aim was to investigate the effect of dehydration on coughing in asthmatic students aged ≤16 years. A validated questionnaire aimed to investigate their respiratory history and cough incidence was used. Urine samples were also collected for assessing osmolality. Wilcoxon test, the Pearson Chi Square and the Fisher Exact Test were used; *p* < 0.05 was assumed as significant. Valid data were obtained from 305 healthy and 56 asthmatic students. Mean urine osmolality was significantly higher in asthmatic than in healthy students (1012 ± 197.7 vs. 863.0 ± 223.0 mOsm/kg, respectively; *p* < 0.001), particularly in symptomatic asthmatic students (1025 ± 191.6 mOsm/kg, *p* < 0.01). Both the incidence and duration of coughing episodes were directly related to the degree of urine osmolality (both *p* < 0.001). Dehydration affects the prevalence and the duration of a cough in asthmatic students aged ≤16 years. Adequate daily water intake should be stimulated in these subjects in order to contain their basic cough attitude.

## 1. Introduction

A cough is a very frequent respiratory symptom occurring in children and adolescents and can cause a substantial burden in terms of recurrent medical consultations, parental stress, and worries [1]. Water is a basic nutritional factor that is involved in all vital processes of human body and it is also crucial in airway homeostasis [2,3]. Dehydration is a condition caused by a negative water balance, such as when the water loss is greater than the water intake [4]. The daily water consumption of children and adolescents was proved to be frequently insufficient to meet their water requirements. This usual attitude was mostly related to their lower sensitivity to thirst and to missing parents’ recommendations [5,6]. Consequently, children and adolescents are more likely to incur dehydration and a higher urine osmolality [7,8].

Normal hydration has been documented as capable of protecting the airway epithelium and promoting proper mucociliary clearance, [7], while dehydration stimulates the production of pro-inflammatory mediators that affect the airway calibre, particularly in asthma [9].

Although dehydration was only episodically considered in real-life respiratory homeostasis, a recent study proved that a cough is significantly more prevalent even in healthy young students if they are dehydrated [10].

The aim of the present real-life study was to investigate the relationship existing between the hydration status and both the frequency and duration of coughs in asthmatic children and adolescents.

## 2. Materials and Methods

The present study was a monocentric, observational, real-life investigation. The operational tools were the anamnestic questionnaire and the individual measures of urine osmolality. The present sample of asthmatic students was obtained from the overall cohort of young students considered in a previous study [10].

Population: students attending the same primary and secondary school institute (the “Istituto Comprensivo Statale 12”, Bologna, Italy) were invited to participate on a voluntary basis. Exclusion criteria were: (a) subjects aged >16 years; (b) inadequate completion of the questionnaire; (c) insufficient collection of the urine sample; (d) smoking habit by any family member; (e) individuals complaining of eating disorders or intolerances; (f) the presence of relevant comorbidities; (g) lack of parents’ informed consent [10].

**Figure A1:**
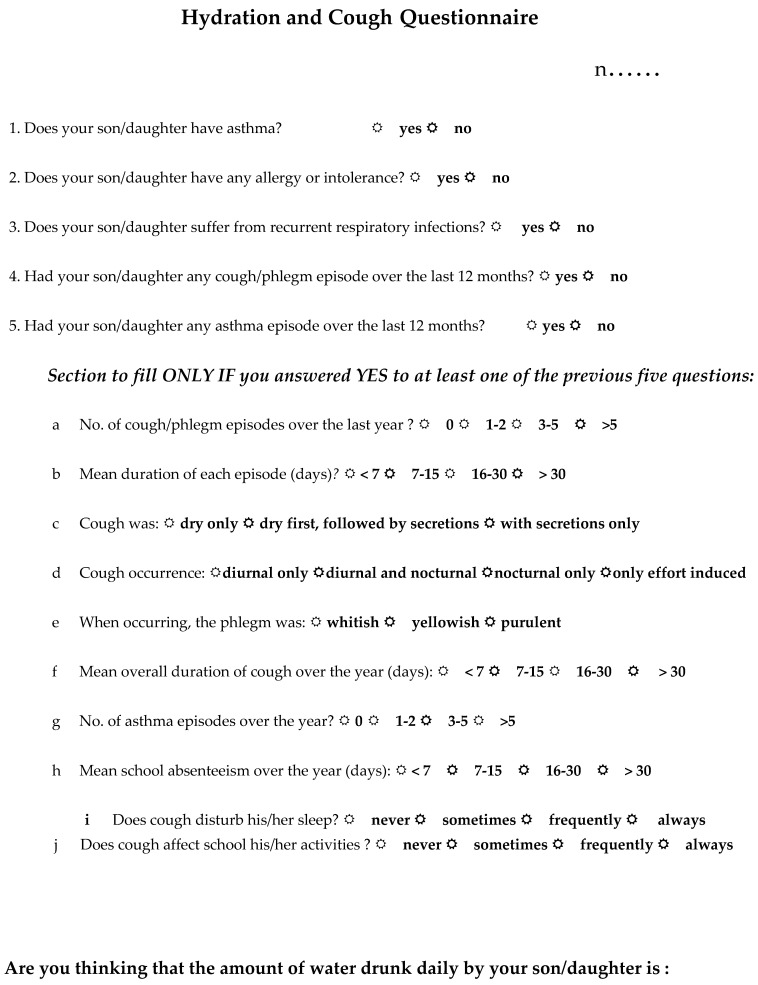

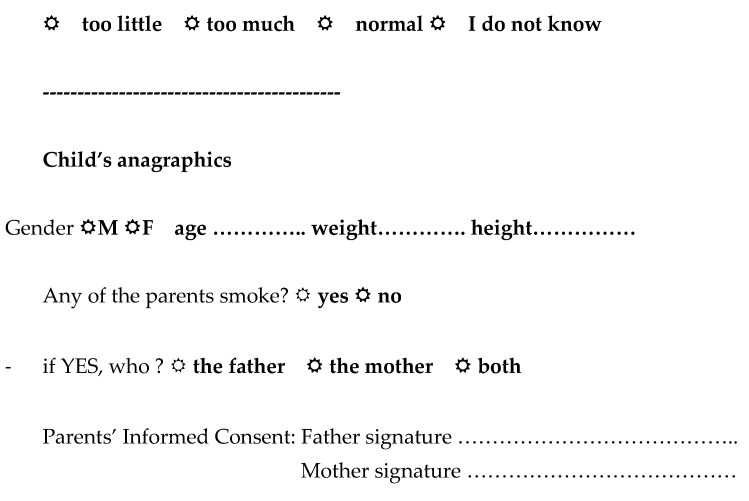
Hydration and Cough Questionnaire.

The questionnaire to be filled in by parents was anonymous and consisted of 22 items ([10]; see Figure A1). It was aimed at checking the presence of asthma, the incidence of cough, and the duration of cough episodes during the previous 12 months.

Urine osmolality: osmolality was measured in urine samples collected 30 min. after breakfast and avoiding the first urine emission in the morning. Urine samples were aliquoted and stored at −20 °C. Measurements were carried out in a centralized laboratory by means of a freezing point depressing osmometer (Osmo 3250, Advanced Instruments, Norwood, MA, USA) [10]. Considering the mean temperatures during spring/summer months in Italy, the hydration status was assessed over a restricted period of the year (February–March 2017) in order to minimize the possible influence of climatic factors on hydration. According to previous studies [7,11,12], osmolality values ranging 800–1000 mOsm/kg were assumed as corresponding to mild dehydration, while those higher than 1000 mOsm/kg as due to high dehydration [13].

Subjects reporting the use of any bronchodilator prn <3 times/month and/or those without any wheezing episodes over the last two weeks before their recruitment were classified as asymptomatic.

Data obtained from the asthmatic sample of students were compared to those of healthy students of the previous study [10].

Statistics: anthropometrics and urine osmolality of healthy and asthmatic students were compared by gender. Osmolality was also compared in students symptomatic and asymptomatic of asthma. Statistics: Wilcoxon test was used for continuous variables, while the Pearson Chi Square and the Fisher Exact Test were used for statistical comparisons. Data were reported as means ± SD, and *p* < 0.05 was assumed as the limit of statistical significancy.

The study was approved by the Ethical Board of AIST (the Italian Association for Study of Coughs) and by the Council of the School Institute (ethical code: 23/CC/2016).

## 3. Results

A total of 400 students were invited. Three-hundred-sixty-one students returned valid questionnaires: 305 were healthy students (male = 155), while 56 claimed to be asthmatic, and then considered for the present study. Of them, 24 were asymptomatic and 32 symptomatic, equally distributed by sex (males *n* = 13 and 16; females *n* = 12 and 15, respectively). Thirty-nine responders were excluded: *n* = 13, claiming relevant comorbidities; *n* = 21 returning incomplete questionnaires;), *n* = 5 missing patents’ informed consent.

Anthropometrics of participants were absolutely comparable and reported in Table 1. Urine osmolality assessed in healthy and in asthmatic responders (*n* = 155 + 56) was then compared.

While the mean (±SD) urine osmolality in healthy students was 837.1 ± 220.2 mOsm/kg (range 148–1293 mOsm/kg), it was 1012.4 ± 197.7 mOsm/kg in asthmatic students (Table 2). In particular, mean urine osmolality was 909.4 ± 190.7 mOsm/kg in asymptomatic, and 1025.4 ± 191.6 mOsm/kg in symptomatic asthmatic students, respectively (*p* < 0.01) (Table 3).

When compared to healthy students, only 14.3% of asthmatics (*n* = 8/56) showed normal urine osmolality, while 28.6% (*n* = 16/56) were mildly dehydrated, and the remaining 57.1% (*n* = 32/56) highly dehydrated. There was a significant difference between healthy and asthmatic subjects, and also between asymptomatic and symptomatic asthmatic students (Table 3). Differently from data previously obtained in healthy students of the same age [10], the prevalence of dehydration in asthmatic students was slightly higher in those aged ≤10 years (26/29) when compared to those age > 10 years (21/27) (OR = 2.48; 95% CI: 0.45–16.88).

The incidence of coughing episodes was equally distributed by gender (*p* = 0.85) and age (*p* = 0.76). Despite the presence of normal urine osmolality, the incidence of coughing episodes was significantly higher in asthmatic than in healthy students (97.5% vs. 62.5%, respectively) (Table 4). This difference proved even more evident in dehydrated asthmatic students (always *p* < 0.001) (Table 4). Moreover, the incidence of coughing episodes was directly related with the students’ claim of asthma and with their dehydration status.

Both in healthy and in asthmatic students, coughing episodes showed a significantly longer duration by their hydration status (*p* < 0.001), even if the incidence of coughing episodes lasting longer than two weeks was significantly higher in asthmatic students (*p* < 0.01) (Table 4).

## 4. Discussion

Two main mechanisms modulate the normal water balance: the thirst sensitivity and the secretion of an anti-diuretic hormone (ADH). These two mechanisms control the homeostasis of plasma liquids up to the 1200 mmol/kg maximal urinary concentration [14,15].

As previously reported, normal urinary osmolality ranges 500–800 mOsm/kg over the 24 h in healthy subjects [14], while values >800 mOsm/kg are regarded as corresponding to the occurrence of mild dehydration, and those >1000 mOsm/kg to the maximum possible urine concentration [13].

Inadequate hydration has already been shown in a large proportion of healthy students aged 6–16 years [10,11,16,17]. The effects of acute or/and persistent dehydration have been described in this age range [13], and proved capable of inducing neurological, cardiovascular and metabolic disturbances [12]; a lower mental and/or physical performance [3]; a significant decrease in attention, and an impaired short-term memory [18,19,20,21,22,23,24,25,26,27,28,29]. This evidence is obviously crucial when considering that all these negative effects can likely affect the students’ school performance, even substantially.

Even if its effects on cough have not yet been investigated in young asthmatic students, dehydration has long been known to worsen respiratory symptoms and lung function in patients with pre-existing respiratory diseases, particularly with bronchial asthma [17]. It should be pointed out that dehydration was described as a condition that is capable of favoring and enhancing histamine release from the mastocytes in bronchial asthma, while Type2-histamine receptors were shown to also be involved in the increase of blood vasopressin that, in turn, contributes to the regulation of the hydration status [23,24,25].

It was also proved that appropriate hydration is a crucial condition in other respiratory conditions as it can contribute greatly to preserving the proper environment of the airway epithelium, such as the conditions required for effective mucociliary clearance [22,27,28,29,30]. On the other hand, hyperosmolality of the airway surface lining can induce substantial damage and disruption of the tight junctions embedded within the airway epithelium, thus further exposing cough-mediating airway receptors to the effects of exogenous stimuli [31].

Data of the present study provided the first evidence on the real-life relationship existing between the dehydration status and coughing in asthmatic students aged ≤16 years, particularly in symptomatic individuals. To point out that the greater effect of dehydration on coughing observed in younger asthmatic students can be likely explained (or at least partially explained) by their relatively wider body surface that allows a stronger perspiration, particularly during sustained exercise (such as in usual conditions for this age range) [19,21].

The present investigation has some limitations [32]. First, the study is a monocentric survey carried out on a limited cohort of subjects; second, the reports on coughs are based on parental reports; third, the precise mechanisms underlying the cough were not investigated because the study was only aimed at the preliminary real-life assessment of cough incidence in young asthmatic students. The study also has some points of strength, in our opinion, such as: the homogeneity of the study population, the use of a validated questionnaire, the restricted observational period, the availability of careful biological measurements, the adoption of appropriate statistical methods.

In conclusion, the results of the present study suggest a clear relationship existing between coughing and real-life hydration status in young asthmatic students. Water consumption should then be highly implemented and stimulated in these asthmatic subjects in order to easily minimize, at low cost, their basic coughing liability that can frequently limit their quality of life.

## Figures and Tables

**Table 1:** Anthropometrics of healthy and asthmatic students by gender (means ± SD).

	Healthy Males (*n* = 155)	Asthmatic Males (*n* = 29)	Healthy Females (*n* = 150)	Asthmatic Females (*n* = 27)
Age (years)	11.6 ± 3.8	11.4 ± 4.1	11.9 ± 3.7	11.5 ± 3.9
Weight (kg)	39.7 ± 13.4	39.2 ± 12.9	38.1 ± 11.6	38.6 ± 12.0
Height (cm)	146.2 ± 16.0	145.8 ± 16.6	134.1 ± 11.0	135.2 ± 11.4
BMI	18.1 ± 3.2	17.9 ± 3.8	16.8 ± 2.6	17.1 ± 3.6

**Table 2:** Mean urine osmolarity in healthy and in asthmatic students (means ± SD), and the corresponding % prevalence of different hydration status. While osmolality values ranging 500–800 mOsm/kg are normal, values > 800 mOsm/kg correspond to mild dehydration, and values > 1000 mOsm/kg correspond to severe dehydration.

	Healthy Students (*n* = 305)	Asthmatic Students (*n* = 56)	*p*
mOsm/kg	837.1 ± 220.2	1012.4 ± 197.7	0.01
% Prevalence			
Normal osmolarity	120 (39.3%)	8 (14.3%)	0.001
Mild dehydration	86 (28.2%)	16 (28.6%)	0.08
Severe dehydration	99 (32.4%)	32 (57.1%)	0.001

**Table 3:** Mean urine osmolarity in asymptomatic and in symptomatic asthmatic students (means ± SD), and the corresponding % prevalence of different hydration status.

	Asymptomatic Asthmatic Students (*n* = 24)	Symptomatic Asthmatic Students (*n* = 32)	*p*
mOsm/kg	909.4 ± 190.7	1025 ± 191.6	0.01
% Prevalence			
≤800 mOsm/kg	6 (25.0%)	2 (6.2%)	0.001
>800 mOsm/kg	18 (75.0%)	30 (93.8%)	0.01

**Table 4:** Relationship between hydration status and cough by frequency and duration of coughing episodes in healthy and asthmatic students. Regressions for each subgroup with different osmolality.

	Healthy Students (*n* = 305)		Asthmatic Students (*n* = 56)	
	≤800 mOsm/kg (*n* = 120)	>800 mOsm/kg (*n* = 185)	≤800 mOsm/kg (*n* = 8)	>800 mOsm/kg (*n* = 48)
Cough episodes/year (*n*)				
1–2	117 (97.5%)	91 (49.2%)	5 (62.5%)	17 (35.4%)
3–5	3 (2.5%)	67 (36.2%)	2 (25.0%)	21 (43.8%)
>5	0	27 (14.6%)	1 (12.5%)	10 (20.8%)
*p*		0.001		0.001
Duration of episodes (days)				
<7	103 (85.8%)	136 (73.5%)	5 (62.5%)	25 (52.1%)
7–15	17 (14.2%)	41 (22.2%)	2 (25.0%)	19 (39.6%)
16–30	0	8 (4.3%)	0 (12.5%)	4 (8.3%)
*p*		0.001		0.001

## Data Availability

Authors do not wish to share their data without their permission.
